# Feasibility of a mobility programme for people with dementia in the respite care setting: results of the DESKK study

**DOI:** 10.1186/s12877-020-01728-z

**Published:** 2020-09-07

**Authors:** Steffen Heinrich, Caren Horstmannshoff, Bernhard Holle

**Affiliations:** 1 University of Applied Sciences - St. Gallen, Institute for Applied Nursing Sciences, Rosenbergstrasse 59, 9001 St. Gallen, Switzerland; 2 Technical University of Applied Sciences – Rosenheim, Hochschulstrasse 1, 83024 Rosenheim, Germany; 3 German Center for Neurodegenerative Diseases, Site Witten, Stockumer Strasse 12, 58453 Witten, Germany

**Keywords:** Dementia, Respite care, Mobility, Exercise, Preferences

## Abstract

**Background:**

Caring for people with dementia (PwD) is often challenging for caregiving relatives. Respite care (RC) is a commonly used short-term inpatient service. The provision of RC can serve as a link between home care and institutional care and can help to stabilize the care provided at home. During RC, the everyday functional skills of PwD can be improved or stabilized through systematic mobility training. However, no specific mobility programme exists for this setting. The aim of the DESKK study was to develop and test a mobility training programme for PwD in the RC setting in Germany.

**Methods:**

A quasi-experimental pilot study was conducted in a specialized RC centre for PwD. Qualitative and quantitative data were collected and analysed using a mixed methods design.

**Results:**

The DESKK mobility programme may be introduced in the RC setting depending on the required time and professional resources. The mobility programme had a high acceptance rate among the staff involved. Ongoing documentation of the mobility exercises were challenging. During their stay (2–4 weeks), the physical function level of the included PwD (*n* = 20) increased regarding leg strength, gross motor coordination, fine coordination of the fingers and hand strength.

**Conclusions:**

The DESKK mobility programme showed a high acceptance rate by the staff and was usable in daily care routine for the most part. These aspects indicate that the programme has the potential to be successfully implemented in the RC setting. The DESKK concept is described in the form of a practice-friendly website to facilitate its use in clinical practice after its successful evaluation.

## Background

Globally, caregiving relatives play the most important role in supporting people with dementia (PwD) at home [40]. Therefore, it is very important to support caregiving relatives in this task.

In an optimal situation, the caregiving relative would have time to rest to relieve the burden associated with caregiving during a so-called “respite care” (RC) stay of his or her care-dependent relative.

Internationally, there are a considerable number of different structured RC facilities, ranging from ambulatory household care support to inpatient facilities and from professional to honourary structured providers. In Germany, RC is a short-term inpatient service for care-dependent persons that can be used for a period of time (between four to eight weeks). RC was established to support caregiving relatives in the event of problems related to providing stable care arrangements at home [36]. RC centres have an average growth rate of 6.5% per year (2003–2015), providing support for over half a million care-dependent individuals in 2015 [5]. During this short-term stay, the care-dependent person is supposed to obtain optimal care and support for his or her return home. Important requirements for a successful return home are sufficient mobility function capabilities to perform activities of daily living (ADLs). Physical exercise training is important for performing ADLs [13]. However, no evaluated physical exercise programme exists for the RC setting. This is not only true in Germany but also internationally [26].

A mobility programme for RC centres must address specific aspects associated with this setting. RC centres provide short-term stays for care-dependent individuals; thus, there is only a short period of time available for structuring suitable mobility programmes. Furthermore, a successful mobility programme should also focus on the time after the RC stay, offering the possibility of ongoing training. As in all inpatient care settings, the number of care-dependent PwD has increased in recent decades; it is expected that more than 50% of the care-dependent residents in inpatient care centres have cognitive decline and/or dementia [18, 34]. For this reason, a mobility training programme in RC centres should also provide opportunities to include people with cognitive decline and/or dementia. Accordingly, the development and initial test of the mobility programme is focused on this population. This project was carried out along with the DESKK study (Dementia-specific RC Concept).

The main aims of the DESKK study are the development of a mobility programme and the performance of a pilot test to assess the usability of the programme for care-dependent persons in dementia-specific RC centres. Another component of this study is the development of a tailored counselling programme for the caregiving relatives of care-dependent individuals. The results of this parallel counselling programme within the DESKK study will be addressed in another article (in preparation). More detailed information about the two study components is included in the study protocol [17].

This paper discusses the findings of the formative evaluation of the DESKK mobility programme, including barriers and facilitators to implementing the programme. In addition, findings related to changes in the residents’ mobility levels (physical functioning) during their RC stay are presented (summative evaluation). The following research questions are answered in this article:

1. *Formative evaluation:*
  How acceptable was the programme from the perspectives of RC centre staff?
2. *Summative evaluation:*
  What changes in the mobility function levels of the PwD occurred during the DESKK mobility programme intervention?

## Methods

The methods for developing and testing the DESKK mobility programme, the intended data collection procedure and the instruments that were used are described in detail in the study protocol [17]. The trial is registered at trials.gov (NCT03578861).

### Study design

DESKK was a pilot-based, quasi-experimental feasibility study that included a formative evaluation. The intervention was performed as a single-centre study at a dementia-specific RC centre.

### Sample

It was planned to obtain data of 30 PwD and their caring relatives. Furthermore, RC staff were included to gain data for the formative evaluation.

### Setting

The study was carried out in a dementia-specific RC centre with a ten-person capacity (common size for an RC in Germany) in the state North-Rhine-Westfalia, Germany. The dementia-specific RC was built as a pilot facility in the RC setting in 2016. The RC is an independent subunit in a long-term care centre called Haus St. Antonius Paderborn.

The infrastructure of the RC where this study took place was already partially adapted to the specific needs of PwD. For example, there were no visual barriers on the ground, and a circadian light system was installed to support the physical diurnal rhythm of PwD.

Furthermore, the care assistants in this RC performed basic mobility rehabilitation processes with some of the RC guests, but these processes were not systematically integrated in the daily care routine and were not based on assessment.

### Inclusion criteria

Only dyads of PwD and their caregiving relatives were planned to be included in the DESKK intervention. This was performed to analyse potential effects of the mobility programme focused on the care-related burden of the caregiving relatives.

#### PwD

- Willingness to participate in the mobility programme (formalized informed consent explained by a nurse [signed by the caregiving relative if necessary])
- Capability of understanding and following the training instructions with the support of the training coordinator (subjectively rated by an experienced RC nurse)
- Capability to stand and walk short distances (minimum of three metres) with the support of the training coordinator
- Minimum stay of two weeks at the RC centre

#### RC staff

The RC staff participated in the DESKK study to assess programme usability and acceptance (formative evaluation). The following inclusion criteria were defined:

- Willingness to participate in the DESKK study (informed consent)
- Suitable German language skills

### DESKK mobility assessment

The mobility function level of PwD is the primary outcome of the summative analysis. Mobility function was measured by the DESKK mobility assessment. This assessment battery consists of the following physical function tests: the Short Physical Performance Battery (SPPB) [15], the Box and Blocks Test (BBT) [28], the Strength-Dexterity (SD) Test [1] and the Nine-Hole Peg Test (NHPT) [28]. These tests have already been used in the German context, and the necessary information to conduct the tests is also available in German [2, 6, 31].

The DESKK mobility assessment was used to obtain empirical evidence based on the assessment scores. Based on these scores, direct training recommendations for the RC staff were developed. For these recommendations, a cut-off score was set for each area that was tested for mobility function. These cut-off scores were developed based on the literature regarding the utilized assessments. However, reference tables for these assessments were only available for seniors without dementia [1, 15, 16, 28]. For this reason, the assessment scores for seniors with “weak” physical abilities were used as reference cut-off scores in the DESKK mobility assessment. Weak was defined as the weakest third of the tested sample related to the functional abilities of the tested areas in the group of individuals aged 80 years or older.

The decision to set the cut-off-scores this way was based on the literature evidence about significantly weaker ADL abilities and a higher risk of falls in these individuals than in elderly people without dementia [32]. Scores beneath the cut-off thresholds were rated as 0, and scores above these levels were rated as 1. A score of 0 implied that training for that specific tested area was necessary, and 1 implied that physical training for that area was optional. The assessments and their cut-off scores are displayed in Table 1. After a pretest with 6 PwD before the start of the intervention, the cut-off scores for the strength-dexterity test were lowered to the weakest 25% of the population in the sample of Alcenar [1] because of a noticeable lower mean hand strength of the tested PwD.

**Table 1:** Cut-off scores for the DESKK mobility assessment

Body area	Assessment	Mobility function	Cut-off scores to receive 1 point
Upper Limb	BBT	- Gross motor coordination and arm strength	> 30 blocks per minute (male) > 35 blocks per minute (female)
	SD	- Hand strength	> 20 kg (male) > 13 kg (female)
	NHPT	- Fine coordination of the fingers	< 35 s to complete task
Lower Limb	SPPB	- Leg strength - Balance - Gait coordination	< 20 s (chair rise 5 times) > 9,9 s semi-tandem stand < 6.52 s (3 m walk)

The secondary outcomes “cognition” and “behavioural symptoms” were assessed by the Mini-Mental State Examination (MMSE) [12] and the Nurses’ Observation Scale for Geriatric Patients (NOSGER) [37] for a description of relevant study population characteristics and necessary abilities to perform the mobility programme.

### DESKK exercises

The DESKK mobility exercises were developed based on existing literature [10, 21, 42] and were discussed with the RC staff and external scientists. By the start of the intervention, 16 individual exercises were included along with an exercise poster. Furthermore, an exercise manual was created, in which the exercises on the poster were described in detail, including cognitive tasks and potential exercise (easier vs. harder). These exercises are specialized to train one of the six mobility function areas (see Table 1). Each mobility function area consists of two to five different exercises, which could be chosen by a schooled RC nurse who functioned as rater to create an individualized training programme. Before the intervention started, the German Center for Neurodegenerative Diseases (Deutsches Zentrum für Neurodegenerative Erkrankungen, DZNE) scientists performed the rater schooling. Nurse assistants were not allowed to perform the mobility tests and exercise compilation. However, they were schooled to perform the compiled exercises written on a training card (for further information, see: www.deskk.info) with the RC guests in the facility. The empowerment of care assistants to perform the DESKK exercises during the daily care routine is a core component of the mobility programme because of limited resources of registered nurses. The exercises for the specific functional areas are all different (for individual training preferences) but were designed to train similar muscle groups. To document the daily exercise routine, a semi-standardized training protocol was developed. Furthermore, mobility courses were installed on a floor of the RC centre, including training devices for spontaneous, short-term physical training of PwD led by nurses or care assistants during regular walks inside the RC centre (e.g., from the bathroom back to the recreation area). The aim of this installation was to create a low-threshold way to encourage additional training of DESKK mobility exercises.

### Formative evaluation of the DESKK mobility programme within the RC Centre

The developed DESKK programme was tested in the RC centre from October 2017 until September 2018. Four group interviews were conducted during the intervention phase at intervals of 6 to 10 weeks to obtain data for the formative evaluation. The time intervals were extended during the intervention period. In the group interviews, the intervention processes, regarding barriers and facilitators, were discussed with the involved RC staff. The interviews were held with the help of a self-developed semi-structured interview schedule (attached as supplementary file) that is based on parts of the Consolidated Framework for Implementation Research (CFIR) model [8]. The framework can be used modularly, i.e., in individual parts, since not all CFIR constructs fit every study design [8]. To select the appropriate CFIR constructs, they were first examined for their relevance and suitability for the study design, the intervention and the setting (primary “inner setting”), based on given CFIR literature [24].

The construct areas were chosen to reflect the structural and personal requirements (with their linked processes) to implement the programme as intended. The chosen construct areas are displayed in Table 2.

**Table 2:** CFIR areas used for the interview analyses

CFIR construct	Construct area
OUTER SETTING	Patient Needs & Resources
	Cosmopolitanism
INTERVENTION CHARACTERISTICS	Complexity
	Adaptability
	Design Quality and Packaging
	Relative Advantage
INNER SETTING	Readiness for Implementation
	Available Resources
	Structural Characteristics
	Access to Information
PROCESS	Reflecting and Evaluating

The group discussions were documented with audio recordings, which were transcribed afterwards. In addition, seven single interviews were conducted using the same method with selected RC staff members, who also took part in the group discussions.

This was done to obtain a deeper insight into individual ratings regarding the project components than would be possible in group interviews because of potential hierarchical influences between the interview participants. Furthermore, a short questionnaire that included quantitative structured, ordinal scaled items was completed by the RC staff to provide the opportunity to reflect changes in essential aspects of the acceptance of the programme over time.

### Qualitative interview analysis

Based on the interview schedule, which was structured on the chosen CFIR constructs, a code tree was created with a deductive procedure using a structured content analysis according to Mayring [27]. Thereby, the interview transcripts were coded by two independent scientists. The material was divided into content sections and codes. Through a circular process, the statements of the interviewees were reduced to the essential content. The coding and analysis processes were performed with the help of MaxQDA 13 qualitative analysis software [41]. At regular intervals, the codes and the coded material were synthesized during a discussion process between the participating project scientists.

### Summative evaluation of the mobility programme in the RC Centre

The DESKK mobility assessment was used to test the mobility function levels of PwD at the beginning of the training period in the RC centre (t0), after two weeks (t1) and, if possible, after three (t2) and four weeks (t3) of their stay. The training schedule was planned with a minimum of 20 min of active training per day. The mobility function levels of the included PwD and the changes over time between t0 and t1 were analysed using SPSS 21 (IBM Corp., Armonk, NY, USA). In addition to descriptive statistics, a Wilcoxon-Mann-Whitney significance test was performed to identify changes in the mobility function level of PwD between t0 and t1.

## Results

### Formative evaluation of the mobility programme in the RC Centre (research question 1)

The data displayed below were obtained from the single and group interviews with the RC staff. An overview of the interviewee characteristics is provided in Tables 3 and 4.

**Table 3:** Interviewee characteristics (*n* = 11)

Characteristic	Results M ± SD n
Age (years)	39.1 ± 12.8
Sex	
- Female	11
- Male	0
Occupational qualification	
- Registered nurses	4
- Care assistants	7
Occupational expertise (years)	5.7 ± 4.5
Works in other care units in addition to the RC centre?	
- Yes	5
- No	6

**Table 4:** Conducted interviews (n = 11)

Conducted Interviews	Number of Participants
Single person (n = 5)	Registered nurses (n = 3)
	Care assistants (n = 2)
Group (n = 4)	Registered nurses (*n* = 4)
	Care assistants (n = 7)
- 6 to 11 participants per group interview	

The following themes were structured based on the CFIR content areas and their definitions:

#### Outer setting

##### Patient needs and resources (extent to which patient needs are known and prioritized)

The needs of the PwD were reflected by the RC staff. In conclusion, it was noted that the *needs* and their resources were *very different* depending on the specific PwD: “Some (PwD) like the socializing aspect of the common room in the RC centre, but others find it stressful. Most of the guests like that they can act their own way and that they are not judged.” [S-MA7 (103)]

“What the guest’s wishes and dreams are is, of course, very deeply associated with the level of dementia.” [S-MA5 (14)]

As an important factor for the wellbeing of PwD, it was noted by the RC staff that PwD could experience pleasure during their stay: “The enjoyment of the guest should always be made the centre of attention.” [G1-(216)].

##### Cosmopolitanism (degree to which an organization is networked with other external organizations)

There were *no formalized collaborations* of the RC staff mentioned, regarding relevant organizations, which are connected to physical training programmes for PwD. One registered nurse mentioned: “Some of the guests get physical therapy from external providers, but we are not really connected to them.” [G1-(176)] The persons included in the mobility programme received no physical therapy during their stay.

#### Intervention characteristics

*Complexity (perceived difficulty of implementation - > How complicated is the intervention?)*

The mobility exercises were rated as *self-explanatory in most cases*. If questions arose, the exercise manual could help the staff to answer them: “I (RC staff) look inside (the manual), and it works for me and my colleagues. It’s good that requirements for a successful therapy session are described once more in detail.” [G1-(101)].

Nevertheless, *some complex exercises* (such as using the Wii®) were not as well accepted by the RC staff as easy-to-use exercises, such as simple ball games: “The thing with the Wii® is that it always depends on who (RC staff) is there. Sometimes it is also not conducted. It’s too complex for some colleagues.” [S-MA7 (45–47)].

The complexity of the mobility assessment was noted as suitable for the intervention. The execution of the assessment took *significant additional time at the beginning* of the study: “If you have done it (the assessment) one or two times, it is in your head […] you need extra time, but the actual process is not very complex.” [S-MA12 (49–51)].

The Box and Block Test was rated a few times as cognitively overwhelming for PwD [G2-(21)].

##### Design quality and packaging (excellence in how the intervention is bundled and presented)

Related to this aspect, no further information could be gathered other than that the presentation and packaging of the mobility programme was *suitable and appropriate*: “Everything alright…” [S-MA5 (87)] “Well, no it’s good I think…good, no problems.” [G1-(132)].

##### Adaptability (degree to which an intervention can be adapted to meet local needs)

Related to the DESKK mobility assessment, it was noted that it “…can be performed in a playful, individual way, which I think is important to increase their (the PwD) emotional accessibility.” [S-MA12 (53)].

Integrating the *physical preferences of PwD* to the exercises was reviewed as a good approach, but with *limited* informative *value* for the RC staff. The mobility preferences were provided by the caregiving relatives in most cases, mainly because PwD were no longer aware of their preferences. There was a lack of detailed descriptions of the preferences of PwD from their caregiving relatives: “Often it is only mentioned that the overall mobility should increase as well as the ability to climb stairs.” [S-MA7 (83)].

##### Relative advantage (stakeholders’ perception of the advantage of implementing the intervention)

It was mentioned that the DESKK mobility assessment provided *valuable information regarding which exercises needed* to be performed: “Well, the assessments filter out what is necessary, and I find it good that specific exercises are recommended. I do not have much time, so it (the mobility training) has to work quickly and effectively.” [S-MA12 (141)].

Furthermore, the *planning* of care rehabilitation was noted to be *accurate based on the information* extracted from the DESKK mobility assessment: “I find it to be persuading, and you get much more contextual information for a good care plan.” [S-MA10 (147)].

Positive effects in PwD were noted by the RC staff: “The (physical) support increased. We do not only sing and clap with our hands anymore, but we are also doing sport activities. That makes a huge difference.” [S-MA7 (120)]

“You don’t need to think about yourself so much. They (the exercise recommendations) are given automatically (by the mobility assessment).” [G1- (376)]

“The advantage is that now you have more exercises that you can do with other people in groups. For example, these balls. This is good for everyone.” [S-MA10 (93)]

“The colleagues see the progress of the guests (PwD) as a result of the (mobility) programme.” [S-MA7 (64)]

#### Inner setting

##### Readiness for implementation (indicators of organizational commitment to implement interventions)

Initially, the DESKK mobility assessment took approximately 30 min per person [S-MA12 (50)]. After six weeks, the time demand decreased to approximately 20 min. After three months, the time required for the performance of the SPPB, BBT, NHPT and SD assessments took approximately 15 min. After 6 months, the time decreased to approximately ten to twelve minutes [G4 - (120)].

In general, *most* of the chosen *mobility exercises received positive feedback* from the RC staff two months after the mobility programme began. In particular, the ball-related exercises in groups (arms and/or legs) were noted as being effective: “Even people who are crooked can catch a ball, and they do!” [S-MA6 (42)]. “Yesterday and the day before we played ball games […]. Everybody stood up, put the ball above, and lowered the ball underneath, with feet, with hands. We all had fun and laughed.” [G1 - (130–131)].

Furthermore, the use of a pedometer together with a bicycle simulation video (DVD) generated high motivation levels in PwD: “Before (the DESKK mobility programme), we didn’t use the pedometer. It just stood there. But now, magnificent. We really had days where he (PwD) sat for 1.5 hours and we couldn’t get him to move.” [S-MA10 (98–29)]. “What we often do is use the pedometer.” [S-MA10 (45)].

Four exercises of the DESKK mobility programme were replaced during the intervention phase. The changes took place as a result of the feedback from the RC staff during the group interviews. Exercises that were related to former work objectives of PwD were sometimes not well accepted by PwD. For example, a workbench exercise was only slightly appreciated by PwD and therefore was excluded after two months: “That was often the case, especially for men. If they had to work all their lives in a handcraft-based job, then they connect it in the way: ‘Why should I do this here again?’” [G2–1 (301–302)]. The new exercises (e.g., bowling, or an easy form of playing tennis in a seating position) for the different physical function areas were also created based on the RC staff suggestions. Some care assistants applied these exercises sometimes before the DESKK mobility programme started but were not considered in the first version of the DESKK exercise programme.

The registered nurses noted a *high staff fluctuation within the RC centre*: “It is important that the employers are constantly there (in the RC) and can guide the guest through the mobility programme.” [S-MA7 (26)]. “I think this is sad; it should be applied with more continuity. There is too much personal change […] Why is it not possible, only integrate (DESKK) trained colleagues into the RC?” [S-MA5 (26)]. Because of staff fluctuations, *some of the RC staff were not sufficiently familiar with the mobility programme* and could not perform it as desired: “Two colleagues (registered nurses) are jumping between the wards and don’t properly know how to apply and document the programme because they do it too seldom and irregularly.” [S-MA7 (95)].

The overall self-rating of the RC staff concerning their subjective feeling of being familiar with the DESKK mobility programme increased during the intervention period. When they were asked for their confidence level during the execution of the programme in percentage form, the answers of the involved staff ranged from 60 to 100% after 6 months of the intervention. [G4-(289–328)].

##### Available resources (resources dedicated for implementation and ongoing operations)

Mobility programme execution

The personal *time and skill resources* of the RC staff were *sometimes noted to be challenging* for the execution of the mobility programme as intended: “If there are two care assistants, you can do much, much more. It also depends on who is on duty (how familiar is he/she with the mobility programme).” [S-MA7 (16)].

Some nurses noted a lack of time resources to perform the individual exercises: “Sometimes I don’t have the time to train with the guests together or even one after another. I have to perform the basic care…I cannot go in the garden like the care assistants.” [S-MA10 (15)].

It was mentioned that a *separate room would be an advantage* for facilitating the individual exercises, especially if there is too much noise and general unrest in the common room where the exercises were usually performed: “It was already mentioned prior to this that they (the PwD) quit (their exercises), because there is too much noise and unrest (in the common room) for them.” [G1-(215)].

Related to the mobility course, these problems did not seem to occur: “The mobility course is not a problem. I can perform it directly after usual care.” [S-MA12 (41)]. “I like the mobility course in general. They (the PwD) get active, and I can integrate it into my daily routine.” [S-MA5 (19)].

Mobility programme documentation

The *documentation* of the exercise in the training protocol was also *noted to be challenging by some registered nurses*: “Yes, the last two days I forgot it (the documentation). I remembered it when I sat in my car. There were two admissions today…and then you are just elsewhere with your mind.” [S-MA10 (17)]. As a possible solution for this problem, it was suggested by different RC staff members to integrate the DESKK training protocol into the electronic documentation system instead of performing it with paper and pencil: “It (the training protocol) should be integrated into our documentation system. Then, you have to check it and cannot forget it anymore.” [S-MA6 (39)].

The *documentation process itself was noted as time efficient* in most cases: “The documentation is also quite fast.” [S-MA12 (41)]. “I don’t find it (the documentation) time intensive, and it (the training protocol) is well structured.” [S-MA5 (48)].

##### Access to information (ease of access to digestible information and knowledge about the intervention)

Related to the use of the assessment, the primary assessor noted problems with the data collection process because of the organizational aspects of the centre: “Well, since we started with the project, I tried to deal with it myself. Thereby, (data collection) mistakes occurred, and maybe it was my fault not to mention this and to obtain help.” [S – MA7 (28)].

The *regular exchange between the staff and* the DZNE *scientists* was mentioned to be *adequate* and helpful by most of the RC staff: “I think this (the interviews) is important because DESKK is important. I think it is good to have these discussions.” [S-MA6 (72–74)]. In two cases, more details about the programme were desired: “I would have appreciated knowing more about the DESKK concept in general, not only the mobility programme but also the counselling intervention.” [S-MA12 (95)].

The *exchange and feedback of the nurses and care assistants with the RC managers* were mentioned to be *well functioning*: “I am well supported and if something went wrong (with the execution and documentation of the mobility programme), she (RC manager) gave feedback to the concerned colleagues to change the situation.” [S – MA10 (75)].

#### Programme acceptance over time (quantitative)

**Fig. 1:**
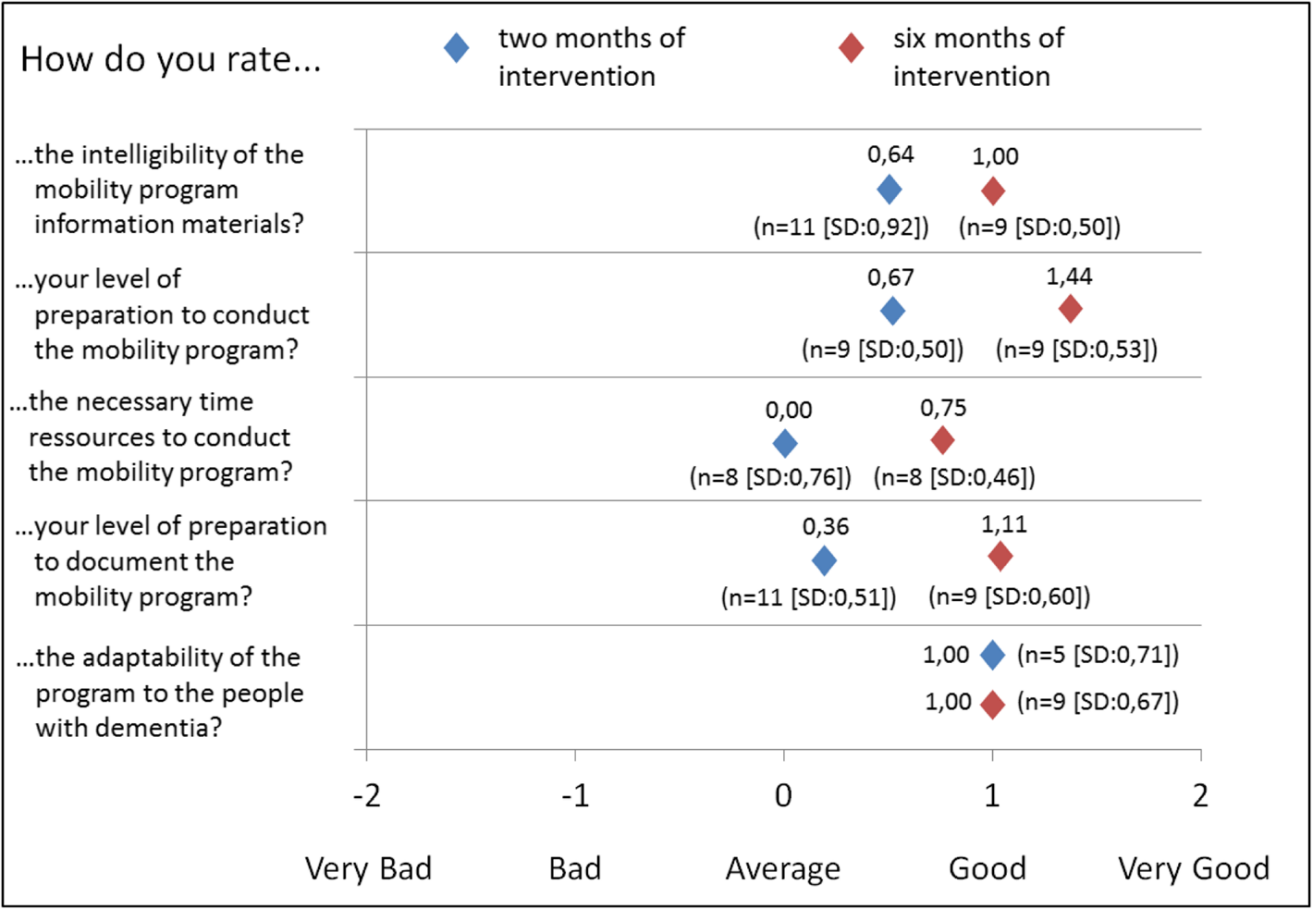
Usability rating of the mobility program by nursing staff between two and six months after the intervention started

The quantitative data from the questionnaires regarding the mobility programme acceptance by the RC staff are shown below in Fig. 1. The data comparison is based on the data from the first group interview, the interview that was conducted two months after the intervention started (t0) and the fourth group interview that was conducted six months after the intervention began (t1) because all DESKK staff members (*n* = 11) took part in these interview rounds. The round two and three interviews only included some of the DESKK staff (*n* = 5, *n* = 4, respectively) for organizational reasons. The staff ratings regarding their subjective feelings of being prepared to conduct and document the mobility programme increased between t0 and t1. Furthermore, the time resources that were necessary for the execution of the programme were rated more positively during this period. The ratings of the ability of the programme to adopt the specific preferences and needs of PwD remained stable.

### Summative evaluation of the DESKK mobility programme in the RC Centre (research question 2)

The average length of stay in the RC centre of the included PwD was 17.7 [SD: 6.7] days. The sociodemographic characteristics of the included PwD are displayed in Table 5. In terms of the sociodemographic data, the study participants had a mean age of 81 years and exhibited moderate cognitive impairments. Approximately two-thirds of the participants were female. With an average score of 15.6 measured by the NOSGER, mild to moderate behavioural symptoms were found in the sample [38]. In particular, apathy and defence reactions were most prominent behavioural symptoms in the sample (*n* = 12).

**Table 5:** Characteristics of people with dementia (*n* = 20)

Characteristics	Results M ± SD n
Age (years)	
Mean (SD)	81.0 ± 6.5 SD
Median (IQR)	80,5 (77.3, 86.0)
Sex (n)	
- Female	13
- Male	7
Marital status (n)	
- Married	8
- Widowed	8
- Divorced	4
Mini-Mental State test score (n = 20)	
Mean (SD)	18.4 (± 3.4)
Median (IQR)	18,5 (15.3, 21.5)
Years since dementia diagnosis (n = 16)	
Mean (SD)	3.6 (± 1.45)
Median (IQR)	4 (3, 4)
NOSGER score - area: behavioural symptoms (n = 12)	
Mean (SD)	15.6 (± 1.10)
Median (IQR)	14 (13, 17)

#### Changes in mobility function level

Because of the heterogeneity in the lengths of stay of PwD, most participants could only be assessed between t0 and t1 (after two weeks). Persons with a slightly longer or shorter length of stay were assessed at the end of their stay. Only four participants reached the t2 data collection point, and only three reached the t3 point. Furthermore, in the four early data sets, the t1 data collection was performed too late (after three weeks or more). Therefore, the t3 data from the regular data sets were aggregated with the data from these four deviant collected data files. Therefore, we obtained the opportunity to analyse the regular data sets between t0 and t1 (*n* = 16) and additionally valuable data sets (*n* = 7) between t0 and the last data collection point, which was an average of 26 days after t0. This data collection point is referred to as tx. Between t0 and t1, leg strength improved slightly while gait coordination remained stable, as measured by the SPPB. For the upper limbs, the gross motor coordination/arm strength (BBT) improved significantly (*p* = 0.006; Wilcoxon-Mann-Whitney-Test [not a normally distributed sample]). Fine coordination of the fingers (NHPT) and hand strength (SD-test) improved similarly, but the differences were not statistically significant. The details are shown in Table 6. Compared with the 14-day sample (t0-t1), the group with an average treatment time of 26 days (t0-tx) exhibited similar changes in the functional areas “leg strength” and “gait coordination.” The median score of the functional area “coordination/arm strength” was notably higher in the t0-tx group than in the t0-t1 group. Because of the small sample size, no significance test was performed for the t0-tx analysis. The results are displayed in Table 7.

**Table 6:** Outcome analyses of the mobility function level tested by the DESKK mobility assessment after 14 days (t0; t1)

Time	SPPB Chair stand, 5 times (seconds)	SPPB 3 Metre walking (seconds)	BBT (Blocks per minute)	NHPT Time to complete the test (seconds)	SD-Test Hand strength (kilograms)
	Median (IQR)	Median (IQR)	Median (IQR)	Median (IQR)	Median (IQR)
t_0_	**20.9** (12.5, 33.3) n = 16	**8.2** (6.4, 10.2) *n* = 16	**20** (14, 24) *n* = 15	**29.7** (22.5, 41.8) n = 16	**25.3** (12.9, 32.9) n = 16
t_1_ (14 days ±2.1 SD)	**17.9** (11.8, 29.4) *n* = 14	**7.9** (5.8, 9.4) n = 16	**25** (17, 29) *n* = 15	**28.3** (22.8, 36.9) n = 16	**26.7** (16.2, 31.0) n = 16
	*(lower score is better)*	*(lower score is better)*	*(higher score is better)*	*(lower score is better)*	*(higher score is better)*

**Table 7:** Outcome analyses of the mobility function level tested by the DESKK mobility assessment after 26 days (t0; tx)

Time	SPPB Chair stand, 5 times (seconds)	SPPB 3 Metre walking (seconds)	BBT (Blocks per minute)	NHPT Time to complete the test (seconds)	SD-Test Hand strength (kilograms)
	Median (IQR)	Median (IQR)	Median (IQR)	Median (IQR)	Median (IQR)
t0	**23.2** (17.7, 29.1) n = 7	**8.4** (5.0, 9.1) *n* = 7	**23** (15, 26) n = 6	**35.1** (28.4, 44.1) n = 7	**21.4** (15.0, 30.2) n = 7
tx (26 days ±3.4 SD)	**18.5** (14.2, 24,7) n = 7	**7.6** (6.5, 9.2) n = 7	**27** (15, 31) *n* = 6	**30,3** (23.2, 39.0) n = 7	**23.6** (16.8, 26.9) n = 7
	*(lower score is better)*	*(lower score is better)*	*(higher score is better)*	*(lower score is better)*	*(higher score is better)*

## Discussion

### Formative evaluation

The qualitative data obtained in this study provided a detailed insight into the processes, barriers and facilitators of the implementation of the DESKK mobility programme into the daily care routine. The analysed dementia-specific structures of the RC centre are not common for RC centres in Germany. However, it is likely that this intervention could be acceptable and beneficial in any RC centre that has the training and capacity to provide person-centred dementia care. Detailed information about the given Ressources in this specific RC centre are displayed on the concept website www.deskk.info.

### Mobility assessment and the MMSE

The time required to perform the DESKK mobility assessment (approximately 15 min after three months of intervention) makes it an efficient instrument compared with other mobility assessments [19].

The connection between assessment scores and suggested mobility exercises is an essential component of the DESKK mobility programme; simple cut-off scores guide nurses to make specific exercise recommendations. There is a great need for such practical approaches in this field, and the related solutions have been inadequate until now [4].

Another essential aspect of the DESKK mobility assessment was the use of objective functional physiotherapeutic measurements instead of questionnaires, as are commonly used in nursing practices throughout Germany and abroad [11, 22]. A main advantage of objective functional measurements compared with questionnaire-based subjective measures lies in the higher validity and reliability of the objective measures. Subjectively rated questionnaires depend on the individual feelings of the rater related to the specific test discipline (e.g., climbing stairs). The related high risk of scoring bias is widely known [14], but nevertheless, objective functional measurements are seldom used in nursing practice. A reason could be that functional measurements are not often incorporated into nursing education and that the functional measurements were developed to be conducted by therapists rather than by nurses [33].

### Mobility programme exercises and programme documentation

Most exercises were well accepted by PwD, but some exercises were not. A workbench exercise was not well accepted, and the same result was seen in an exercise with clothespins (see results). The work duties of this sample seemed to not be associated with positive memories as predicted. The literature promotes connecting the activities of PwD with their former habits/activities [3]. In the field of physical activity, it could make sense to differ between former hobbies and work-related activities. No literature on this topic was found, but it would be an interesting research question.

The result that ball games worked especially well for PwD is not surprising because many people had ball game experiences in their earlier lives, and these experiences were mostly related to leisure activities. This might explain why ball-related games are one of the most commonly used physical exercises in the field of dementia care [3].

The use of the Nintendo Wii® seemed to cause problems for some RC staff members. However, the use of this gaming console in dementia care has already been evaluated in different studies in the caregiving setting, and few problems for nurses occurred as a result of its use [20, 23].

A very important aspect of why some exercises and the daily documentation of the DESKK mobility programme in the training protocol were not always performed as intended was related to an unexpectedly high staff fluctuation during the intervention period. The rate of programme use was low for most of the part-time RC staff, which is a well-known inhibitor of the successful implementation of complex interventions in daily routines [29].

The exercises themselves were rated by the staff as well suited for PwD in most cases. However, it is not clear whether the preferences had a real impact on the motivation of PwD to perform specific mobility exercises. In the literature, the effectiveness of preference-based exercise compilations remains unclear, and further research is needed in this area [39].

### Mobility programme documentation

The training protocol was primarily used for empirical data collection but was also intended to act as an activity protocol for the implementation of the programme. As already pointed out, the documentation of the protocol by staff was mostly fragmented and was occasionally imprecise. Some training sessions were not documented in the paper and pencil protocol, or the exact training time was sometimes missing. As reasons for this behaviour the nurses mentioned that the protocol was a paper and pencil version, but they were accustomed to documenting all daily care routines in the electronic system and thereby forgot to fill out the training protocol sometimes. Based on this feedback, the training protocol was embedded in the electronic documentation system after the end of the study.

### Summative evaluation

The sociodemographic findings are quite similar to findings of many other dementia studies in which PwD participated in mobility programmes [13]. The age range between 75 and 85 years also reflects the largest group of people affected by dementia in Germany [7].

The relatively short time since the DESKK participants were diagnosed with dementia might be explained by the fact that many official dementia diagnoses in Germany occur in already advanced stages of the disease or by the fact that PwD are never officially medically diagnosed and, instead, the diagnosis is just determined by the general practitioner or the caregiving relative [25].

The summative evaluation of the programme took into account the general limitation of the small sample and the very heterogeneous mobility function capabilities of the included PwD. As already noted, the sample was smaller than originally planned (goal of *n* = 30 PwD), but in general, a small and heterogeneous sample (see Tables 5 and 6) is expected and common in feasibility studies [30]. In particular, the high level of heterogeneity in the participants’ physical function areas was intended this way. The inclusion criteria were very broad in terms of the physical abilities of PwD to evaluate the adaptability of the mobility programme to different physical ability levels. Interestingly, the different physical capability levels did not notably affect the programme execution. The basic inclusion criteria ensured that PwD could participate in the DESKK mobility programme. The exercises themselves all included variations to decrease or increase the training intensities to adapt to the individual mobility level.

Because of the chosen study design (feasibility study), the quantitative data can only provide first hints about the impact the DESKK programme may have on function and may have a high risk of bias. Tables 6 and 7 are primarily displayed to show the functional characteristics of included study participants and changes during the intervention time. There is no intention to prove the effectiveness of the physical exercises but rather to evaluate their feasibility in the RC setting for PwD. Indeed, most of the literature-based chosen exercises were previously used and evaluated positively in terms of their general effectiveness in improving the mobility function abilities of PwD [9, 13, 35].

Nevertheless, the initial summative findings for the DESKK mobility programme are positive, especially related to upper limb functionality and considering the short individual intervention time for most of the included PwD.

## Strengths and limitations

Because of the feasibility study design, adaptations and modifications of the DESKK mobility programme were possible during the intervention period to improve its usability. However, because of these adjustments (e.g., replacing exercises, modifying of the “dyad” inclusion criteria), the outcome data are not based on a static intervention process, and the quantitative results should be interpreted accordingly, with caution.

The cut-off scores for the mobility assessment battery that was used had to be chosen based on sample scores of seniors without dementia. Therefore, these cut-off scores are not validated for PwD, and it is not clear whether they were optimal cut-off scores for the mobility assessment. Nevertheless, during the pretest and in the regular intervention phase, most nurses noted that the cut-off scores correlated with their subjective feeling about the training needs of PwD. Nonetheless, an adequate validation of these cut-off scores must be performed before the start of a regular randomized controlled trial. At the moment (March 2020), data collection is in progress in two different RC centres aiming to validate the DESKK mobility programme cut-off scores.

Only dyads (PwD and their caregiving relatives) were included in the study during the first seven months (inclusion criteria) to evaluate the effects of the mobility programme related to the caregiving relatives’ burden. However, the caregiving relatives often did not meet the inclusion criteria (e.g., not primarily responsible for the care at home or insufficient German language skills). Because of this finding, the criterion of including only dyads was eliminated and thereby PwD and caregiving relatives were also included as single study participants. Therefore, an increased number of PwD could be included.

The exact training time spent on the different mobility exercises could not be determined because of imprecise data entries and missing data in the training protocol. This limitation and a possible solution (the integration of the training protocol into the electronic documentation system) were previously discussed in this article.

Furthermore, because of a widespread gastroenteritis infection in the RC centre (staff and PwD), the mobility training programme could not proceed for three weeks during the intervention phase.

## Conclusion

The DESKK study showed that the DESKK mobility programme had a high acceptance rate among RC staff. The mobility programme was performed without meaningful project-related staff resources. It could therefore largely be integrated into the existing daily care routines, and PwD were mostly motivated to participate in the exercises; however, external triggers (from the nurses) were necessary in most cases.

In terms of the implementation requirements, it is very important that there is at least one full-time nurse responsible for the correct implementation and documentation of the programme because high rates of staff fluctuation and part-time workers are strong inhibiting factors for a successful implementation of such concepts.

The mobility level of the included PwD improved overall, and the programme was usable despite a very wide range of physical function abilities of the participants. Thus, the programme seems to be highly adaptable. Follow-up analyses regarding homework programme usability and acceptance as well as the necessary (external) support structures will be reported and discussed in another paper. Until then, the DESKK mobility programme can be accessed without additional costs under www.deskk.info (German language). Moreover, the main parts of the mobility programme on the DESKK Website are expected to be translated into English by the middle of 2021.

## Supplementary information

**Additional file 1.** Interview Guide for Individual and Group Interviews-Process Evaluation DESKK.

## Data Availability

Data will be available based on the informed consent of the participants. The datasets used and/or analysed during the current study are available from the corresponding author on reasonable request.

## Abbreviations

PwD: People with Dementia
RC: Respite Care
ADLs: Activities of daily living
SD: Strength Dexterity Test
BBT: Box and Blocks Test
NHPT: Nine Hole peg Test
SPPB: Short Physical Performance Battery
