# Supplementary material for: Feasibility of a mobility programme for people with dementia in the respite care setting: results of the DESKK study
Source: BMC Geriatr. 2020 Sep 7;20:326. doi: 10.1186/s12877-020-01728-z (PMC7487472; doi:10.1186/s12877-020-01728-z)
Supplement: Supplementary file 1 — Additional file 1. Interview Guide for Individual and Group Interviews-Process Evaluation DESKK. [file 12877_2020_1728_MOESM1_ESM.pdf]

Interview Guide for Individual and Group Interviews - Process Evaluation DESKK

Theme structures (control questions)	Interview Sub-Topics	Content checklist<br><br>**Please ask at Follow-Up in each case:**<br>Changes from the last interview?<br>Reasons?
Outer Setting - **Patient Needs & Resources**<br><br>(What is known about the needs and resources that the target group must have in order to implement the intervention?)	To what extent did the programme components meet the wishes and subjective needs of the target group?	- Necessary requirements, which PwD and relatives have to bring along<br>- Over- or underchallenging factors (barriers/obstacles)<br>- To what extent were the preferences of relatives/PwD considered?
Outer Setting - **Cosmopolitanism**<br><br>(How does the networking of the RC centers look like to the outside world?)	Networking between heath care providers inside and outside the RC center<br><br>Networking between mobility providers inside and outside the RC center<br><br>Networking with other providers	- Which providers internally (which offers)<br>- Which external providers (which offers)<br>- What are the contents of the exchange<br>- How does exchange take place<br>- On which (personnel) level

**Intervention Characteristics - Complexity**<br><br>(How difficult is the intervention; how long will it last; how extensive/multifaceted is it; what steps are required)	Complexity of the Mobility programme	- Implementation of assessments<br>- Structuring of the program<br>- Documentation<br>- Homework programme (structuring + instructions for relatives)<br>- Implementation of assessments<br>- Structuring of the program<br>- Documentation/ - Logging<br>- Monitoring
**Intervention Characteristics - Adaptibility**<br><br>(Is the intervention adaptable to the target group?)	Adaptability of the programme to PwD	- Adaptation to functional status PwD (selection of exercises with reference to the level of difficulty indicated)<br>- Adaptation to preferences PwD<br>- Documentation of the adjustments<br>- Adaptation to the needs and requirements of the Relatives
**Intervention Characteristics - Design Quality and Packaging**<br><br>(Structure and presentation of documents/materials used within the programme)	To what extent are the manuals, instruments and illustrations (e.g. the DESKK poster) adequately designed and structured?	- DESKK print documents<br>- DESKK Homepage<br>- Training map

**Intervention Characteristics - Relative Advantage**<br><br>(What are the advantages and disadvantages of the short-term care concept compared to the previous care situation?)	Reflection of the status prior to the short-term care concept	- Effects on PwD<br>- Effects on personal work as carer
**Inner Setting - Access to information**<br><br>(Are the individual intervention steps ordered in terms of time, personnel and structure?)	Perception of the planning processes<br><br>Perception of the institution's internal communication	- Hierarchy Structures (Control)<br>- defined responsibilities<br>- Planning tools?<br>- Processes for arrangements with superiors and colleagues<br>- Quality circle or similar?
**Inner Setting - Structural Characteristics**<br><br>(How is the RC center structured - special features, transferability)	Structure of the RC center	- structures actually available in the RC centre for programme implementation
**Inner Setting - Readiness for implementation**<br><br>(Are (project) objectives clearly communicated, is the achievement of objectives pursued and is the status of the achievement of objectives monitored?	Communication of the project goals (also regarding implementation)<br><br>Integration into the programme structures<br><br>Feedback	- Exchange meetings (type, scope)<br>- Information coordinators?<br>- Evaluation processes

**Inner Setting - Available Resources**<br><br>(Are personnel, material and time resources sufficiently available to adequately carry out the project?)	Time aspects<br><br>Material<br><br>Skills	- Assessments<br>- Preparation<br>- Implementation<br>- Homework programme
**Inner Setting - Access to information**<br><br>(Is information about the purpose and procedure of the intervention clearly described?)	Staff training<br><br>Manual<br><br>Feedback within the scope of process evaluation	- adequate explanations in the training courses?<br><br>- sufficient depth of content - Contact person?<br>• Comprehensibility<br>• Structure (simplicity)<br>• adequate explanation and Feedback<br><br>- are selection of contents and queries meaningful and helpful?
**Characteristics of Individuals**<br>**- Knowledge & Beliefs about the Intervention**<br><br>(Which attitudes and values do the employees and relatives have towards the RC center concept?)	Project goals<br><br>Project implementation<br><br>Results	- Assessment of the realism of the objectives<br>- satisfactory resource-benefit ratio<br>- Effects on the own nursing activity (e.g. perceived competence or excessive demands)<br>- Transferability to other Care facilities

**Process - Reflecting & Evaluating**<br><br>(How was it done, what was realised, how was its effectiveness, what would have to be handled differently in the future?)	Reflected process description and (interim) conclusion on process application	- Evaluation processes regarding the Implementation<br><br>• what worked, what didn't?<br>• resulting change processes<br>• used tools
